# The Capabilities of Honeycomb Core Structures Made of Kenaf/Polylactic Acid Composite under Compression Loading

**DOI:** 10.3390/polym15092179

**Published:** 2023-05-03

**Authors:** M. A. H. M. Yusri, M. Y. M. Zuhri, M. R. Ishak, M. A. Azman

**Affiliations:** 1 Advanced Engineering Materials and Composites, Department of Mechanical and Manufacturing Engineering, Universiti Putra Malaysia, UPM, Serdang 43400, Selangor, Malaysia; amin.azman@upm.edu.my; 2 Laboratory of Biocomposite Technology, Institute of Tropical Forestry and Forest Products, Universiti Putra Malaysia, UPM, Serdang 43400, Selangor, Malaysia; mohdridzwan@upm.edu.my; 3 Department of Aerospace Engineering, Universiti Putra Malaysia, UPM, Serdang 43400, Selangor, Malaysia

**Keywords:** energy absorption, honeycomb core, kenaf fibre, sandwich structure

## Abstract

This study investigated the capability of honeycomb core structures made of kenaf fibre-reinforced polylactic acid (PLA) composite. Two types of kenaf fibre were used in this study, these being woven kenaf and non-woven cotton/kenaf. Initially, the corrugated shape panel was manufactured using a hot moulding compression method. The panel was then cut into corrugated strips, bonded together using epoxy resin to form the honeycomb core structure, and balsa wood used as their skins. The effects of core height and crosshead displacement rate were investigated. The honeycomb core consisted of 20 mm, 30 mm and 40 mm core heights, and the crosshead displacement rate ranged from 2 mm/min to 500 min/min. Of all the samples, core structure with a height of 20 mm tested at 500 mm/min offered the highest value of compressive strength and specific energy absorption, which were 6.23 MPa and 12.36 kJ/kg, respectively. It was also discovered that the core height and loading rate have significant effects on the mechanical properties of the kenaf/PLA honeycomb core structure.

## 1. Introduction

The demand for green, energy-efficient, sustainable, and eco-friendly materials has reawakened interest in researchers and manufacturers regarding natural fibre composite (NFCs). Natural fibres such as sisal, jute, bamboo, flax, and kenaf have been studied by many researchers over the past few years [1,2,3,4,5]. Furthermore, due to its sustainability and low cost, natural fibre composite has tremendously gained popularity. Thus, the attention has shifted to natural fibre-reinforced composite. For instance, the application of natural fibre composite can be found in numerous fields, such as automotive and aviation as mitigation from synthetic to natural fibre [6,7,8,9]. Vehicle weight reduction, sustainability, and renewable have been the major factors in automotive industries. Wu et al. [6] conducted a study by replacing synthetic fibre, which is a glass fibre sheet moulding compound (GF-SMC), with kenaf fibre impregnated with magnesium hydroxide (MH-NFRC). They found that both tensile strength and specific modulus of rupture of MH-NFRC were 116.4% and 109.0% higher than those of GF-SMC, respectively. There has been a lot of research conducted to push the potential of natural fibre to its best capability as an alternative to synthetic fibre, such hybridization of natural fibre, altering the stacking sequence of the natural fibre, fibre treatment, and combining with matrix materials such thermoplastic and resin. Recently, one of the most studied natural fibres in engineering applications is kenaf fibre [10,11,12]. Moreover, Singh et al. [1] have studied the effect of fibre orientation on the mechanical properties of a jute/carbon/glass hybrid composite. It was found that the hybridization of natural fibre with synthetic fibre improved the shear strength. The combination of jute fibre, carbon, and glass is comparable to the non-hybrid/pure synthetic composite. The shear strength of hybrid composite is 119.10 MPa, while pure glass composite and pure carbon are 99.70 MPa and 135.40 MPa, respectively. However, the application of natural fibre in engineering is still minimal, where mostly the natural fibre is only applied as a minor component. For instance, natural fibre may only be used as a skin or face sheet to a sandwich structure and not as the main core material. Jauhar Fajrin et al. [13] have investigated the use of laminated jute and hemp as an intermediate layer (skin) in hybrid structural insulated panels (hybrid SIPs). It was discovered that the average maximum ultimate load for hybrid SIPs with hemp, jute, and oriented conventional strand boards are 591.50 N, 396.25 N, and 305.75 N, respectively. Furthermore, those two hybrid SIPs can withstand high deflection before reaching the failure load. In addition, hybrid SIPs with a hemp and jute intermediate layer show an improvement in deflection, with 240.49% and 370.25% more than the conventional SIPs.

Today, sandwich structures have gained attention among researchers due to their lightweight characteristic, unique porous structure, best structural performance, and cost savings. Nomex materials are one of the most studied materials in recent times due to their mechanical strengths and performance, especially in sandwich structure technology [14,15]. Yan et al. [16] have carried out a quasi-static compressive test involving Nomex normal honeycomb (E-N1-1), Nomex empty absorbent honeycomb (E-A1-1), Nomex CFRP tube (T-1), Nomex tube-reinforced absorbent honeycomb (T-A1-1), and Nomex tube-reinforced normal honeycomb (T-N1-1). It was discovered that due to the filling of CFRP tubes, the normalised elastic modulus E, peak stress, and energy absorption per unit mass (SEA) of tube-reinforced absorbent honeycomb were all raised by 55.74%, 621.69%, and 327.86%, respectively, when compared to empty absorbent honeycomb. In addition, compared to the total of the individually tested CFRP tube and empty absorbent honeycomb, the specific energy absorption (SEA) of the tube-reinforced absorbent honeycomb increased by 30.34%. Generally, based on previous studies, synthetic fibre composite outperformed the natural fibre in terms of strength and stiffness [17,18]. Nevertherless, natural fibres outperform synthetic fibres in some mechanical properties. Natural fibres have the capability to withstand structure change without developing cracks. There has been interest in optimizing the use of natural fibre in sandwich structures. For instance, Zuhri et al. [19] developed sandwich structures with a square and triangular shape core by using flax fibre. The authors found that the square core structure provides better strength and energy absorbing capability compared to the triangular core structure counterpart. Moreover, Mirzamohammadi et al. [20] fabricated sandwich structures by using 2024-T6 aluminum skin and hybrid jute/basalt core with different configurations. The amount of jute (J) and basalt (B) are varied for each sample. The fabrication of the core was done through a hand layup technique with various percentages of carbon nanotubes (CNTs) being dispersed into the epoxy. They discovered that sample A that consisted of the highest volume of basalt fibre with stacking of basalt/basalt/jute/jute/basalt/basalt (B/B/J/J/B/B) showed higher shear strength at a weight fraction of 0.3% CNTs. Besides, the addition of CNT up to 0.30 wt% helped to increase the flexural strength by improving the adhesion of fibre to the matrix. Dario et al. [21] studied impact behavior of hemp/carbon sandwich structures. They fabricated two main hemp cores with a thickness of approximately 9.93 mm (HD_6P) and 8.76 mm (LD_6P), with carbon fibre used as the skins. Moreover, sample HD_6P consisted of 380 g/m^2^ areal density of hemp fibre, while LD_6P samples consist of 190 g/m^2^ hemp fibre. They concluded that in terms of stiffness behavior, HD_6P is better compared to LD_6P. However, the LD_6P sample was able to absorb more impact energy per unit of area (SEA) in comparison with the HD_6P sample. For instance, at 25 J and 45 J impact tests, the LD_6P sample revealed specific absorbed energies that were respectively almost 82% and 73% higher when compared to the HD_6P sample. Furthermore, Saleh et al. [22] developed a novel double cell wall of square core structure made of flax/PLA composite with a cell size between 20 and 40 mm. The authors discovered that a square core structure with a cell size of 20 mm and filled with F150 foam offered better energy absorbing capabilities compared to the other samples. Core cell size and filled foam core affected the mechanical properties. Moreover, in past studies, there were several popular manufacturing processes to produce composite, such as hand lay-up, the vacuum-assisted resin transfer moulding process (vacuum fusion), and compression moulding [23,24]. However, additive manufacturing (AM) or 3D printing, such as fused filament fabrication (FFF), stereolithography (SLA), or selective laser sintering (SLS), is dynamically evolving as the most advanced and high-potential manufacturing process to manufacture products for various applications due to its high accuracy and design freedom. For instance, fused filament fabrication (FFF) allows production of complex parts without requiring specialized tools or processes [25,26,27,28]. Milosevic et al. [29] have conducted a study to investigate the mechanical performance of hemp and harakeke by implementing fused deposition modelling (FFF/FDM). A few dog bone samples were produced which were pure or unfilled polypropylene (PLA), hemp reinforced PLA at 10 wt %, hemp reinforced PLA at 20 wt %, hemp reinforced PLA at 30 wt %, harakeke reinforced PLA at 10 wt %, harakeke reinforced PLA at 20 wt %, and harakeke reinforced PLA at 30 wt %. They found that fiber content influenced the ultimate tensile strength. The increased fibre content resulted in increased ultimate tensile strengths. Hemp- and harakeke-reinforced filaments both had ultimate tensile strengths of 34 Mpa, while the unfilled PLA gave 22 Mpa ultimate tensile strength. Moreover, a dog bone sample of hemp and harakeke at 20 wt % gave the highest tensile strength, which was almost 49% higher than unfilled polypropylene. Besides, the FDM or FFF technology has moved to manufacturing structural elements. Brischetto et al. [30] have developed polymeric sandwich specimens with the use of PLA and ABS materials. Honeycomb and homogenous PLA are manufactured. Specimens with a sandwich structure embedding a PLA honeycomb core and PLA skin are produced via a single extrude. The mean value for the flexural modulus is 1994 N/mm^2^. The specimen with PLA honeycomb core and ABS skin presents the worst performances with a flexural modulus of only 794 N/mm^2^. The bond or adhesion between skins and core of two different materials is bad since two extruders are required. Thus, the result is a lower performance for the modulus of elasticity because of the replacement of PLA with ABS. Despite of the advantages of additive manufacturing, there are few major disadvantages compared to other manufacturing processes, such as wide-range pre-processing, high costing, and limited choice of material [31,32,33].

It can be concluded there are many factors that can affect the mechanical properties of sandwich structures, such as fibre orientation, fibre configuration, core thickness/height, fibre treatment, and many more [34,35,36,37]. Although many studies have investigated the natural fibre-reinforced composites and have attracted significant attention due to their combined benefits of biodegradability and strength, there has yet to be a complete investigation on the kenaf fibre composite as in a sandwich core structure. In this study, the effect of core height at different loading rates is investigated under quasi-static compression loading.

## 2. Methods and Materials

### 2.1. Material

Two types of kenaf fibre were selected, these being woven kenaf and non-woven cotton/kenaf. The woven kenaf supplied by ZKK Sdn Bhd, Malaysia, comes in the form of 2 × 2 twill with a weight of 200 gsm. The non-woven cotton/kenaf that consisted of 30% cotton and 70% kenaf fibre with 200 gsm of weight was supplied by the Malaysian Timber Industry Board (MTIB), Malaysia. Additionally, PLA thin film (supplied by Guangdong Vision BIOPLA Co Ltd., Guangdong, China) with a thickness of 25 μm was also used to manufacture the composite and to fabricate the honeycomb core. Epoxy adhesives (BBT-7892A and BBT-7892B) supplied by Berjaya Bintang Timur Sdn. Bhd, Selangor, Malaysia, were used to bond the core to skins. Here, the balsa wood which acted as the skins with a thickness of 1 mm was supplied by Patriarch Aunthentique, Malaysia.

### 2.2. Fabrication of Sandwich Structure

**Figure 1:**
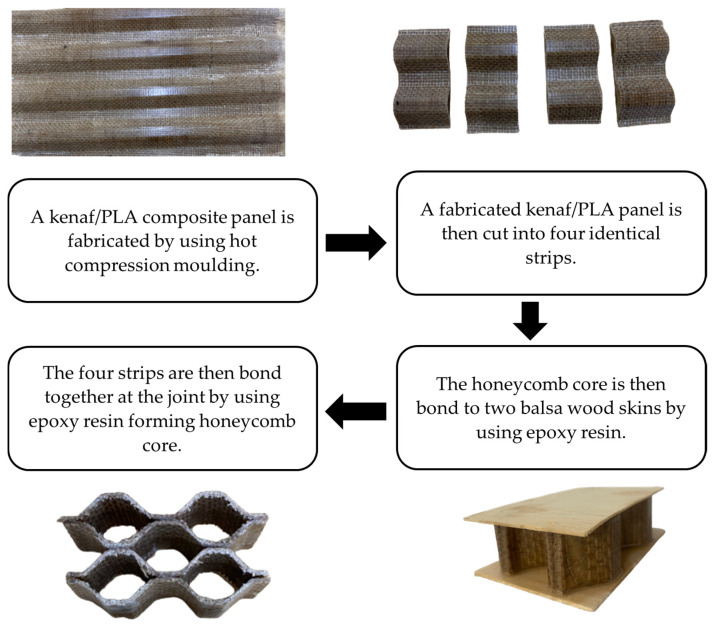
Summary of the manufacturing and fabrication process of kenaf/PLA honeycomb core and the sandwich structure.

**Figure 2:**
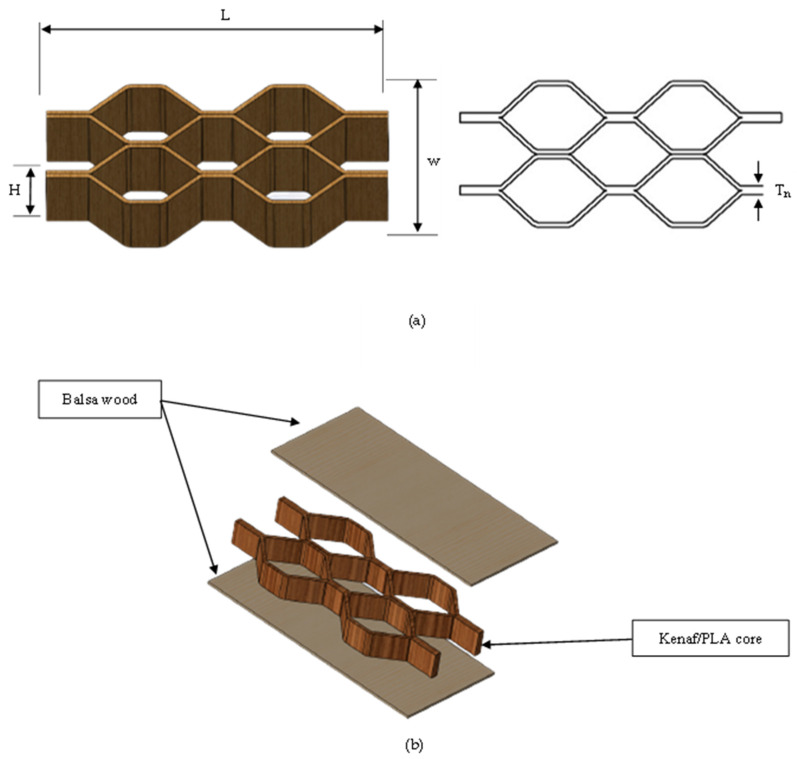
Illustration of (**a**) the core structure and (**b**) fabrication of core to the skin of the sandwich structure.

Here, three different height of honeycomb core structures were fabricated with two different layers, which were 3 and 5 layers. To fabricate the honeycomb core, three different stages of fabrication were involved; (1) manufacturing of the corrugated composite panel, (2) cutting of the panel into corrugated strips by using a bench saw, and finally (3) bonding of the core to the skins by using epoxy resin. At stage 1, the 100 kN hot press machine was used to fabricate the corrugated composite panels. Initially, the woven kenaf and non-woven cotton/kenaf were stacked together in the corrugated mould made from steel. The woven kenaf was arranged as the outer layer and the non-woven cotton/kenaf as inner layer. For the case of 3 layers, two woven kenafs and one non-woven cotton/kenaf with 40 plies of thin PLA film were used. Later, the mould (with a dimension of 300 × 300 mm^2^) was placed into the hot press machine and pressed for at least 7 min under a temperature of 180 °C with a pressure of 30 bar. Once completed, the corrugated composite panel was then cut into small sizes of corrugated strips by using a bench saw machine (stage 2). The corrugated strips were cut into three different heights, these being 20 mm, 30 mm and 40 mm. Finally, at stage 3, the corrugated strips were bonded together to form the honeycomb core structure by using the epoxy resin and later bonded to the balsa wood skin using the same epoxy resin. Figure 1 shows a summary of the manufacturing and fabrication process of the kenaf/PLA honeycomb core sandwich structure. Figure 2 shows the dimensions of the honeycomb core and the completed structure. The details of the final sample product are presented in Table 1.

## 3. Mechanical Characterization

A series of quasi-static compression tests were conducted following the standard of ASTM C365 using the Instron 3382 machine with a load cell of 100 kN. Three different crosshead displacements of 2, 50 and 500 mm/min were selected. Each test was replicated three times, making up 27 samples in total. The sample was placed between the upper and bottom plates and tested until it was completely crushed. The experimental data obtained was in the form of load against displacement. From the graph, a peak load, which indicated the maximum compression stress, was obtained by using formula:

(1) $$\sigma =\frac{F}{A}$$

The energy absorption (*EA*) value was evaluated by calculating the area under the load-displacement curve by using the trapezoidal rule [23]. According to the area under the curve, *E A* was calculated as follows:

(2) $$EA=\int_{0}^{\delta d}LD\delta$$

The energy absorption values were then divided by the mass of the structure to get their specific energy absorption:

(3) $$SEA=\frac{EA}{m}$$

## 4. Results and Discussion

### 4.1. Compressive Behavior of Kenaf/PLA Honeycomb Core Structure

**Figure 3:**
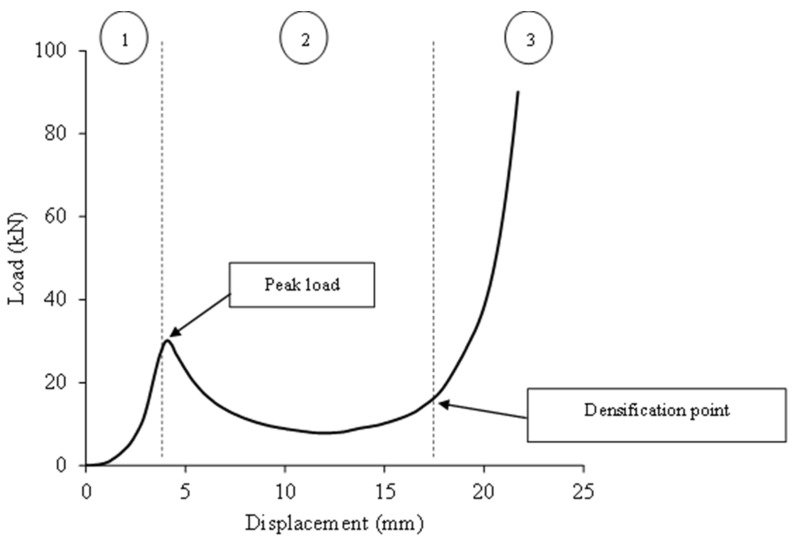
Typical load-displacement curve for L3H20R50 at loading rate of 50 mm/min.

**Figure 4:**
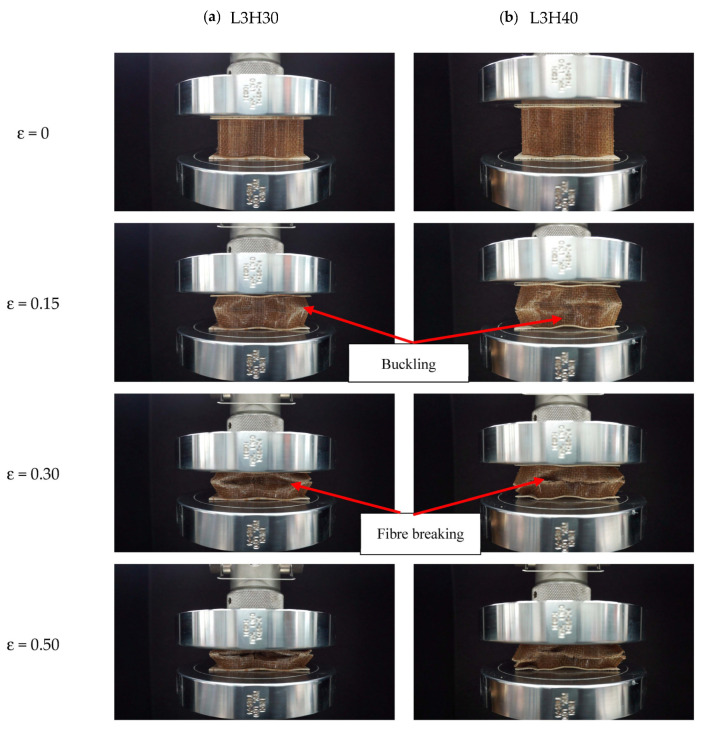
Progress failure modes in the core kenaf/PLA composite structure with (**a**) 30 mm core height and (**b**) 40 mm core height at loading rate of 50 mm/min.

**Figure 5:**
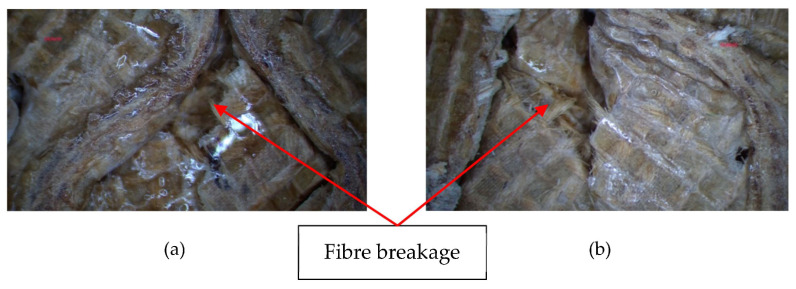
Failure mode of (**a**) L3H30R50 and (**b**) L3H40R50 under microscope.

The plotted load-displacement curve demonstrates the mechanical behavior under the compression loading of sample L3H20R50 at a loading rate of 50 mm/min (Figure 3). The related images that refer to the load- displacement curves are shown in Figure 3. There are three main phases during the compression process, which consist of the elastic (1), plastic plateau (2), and densification region (3). It was observed that there was a small nonlinear response at the very beginning of the compression process due to the non-uniformity of the core initial height, possibly due to the fabrication. As the loading was applied, the force was increased until the structure was no longer able to withstand the loading. Once it reached the maximum point, the structure started to lose its strength and stability, where its change from buckling to fibre broke and resulted in a progressive drop of loading force. Then, in the plateau region, the load was constant until it reached its onset densification point where the structure was completely crushed. Figure 4 shows the deformation of samples with 30 and 40 mm heights, while Figure 5 presents the image of a selected failed sample under an optical microscope.

### 4.2. Effect of Core Height on the Compression Strength

**Figure 6:**
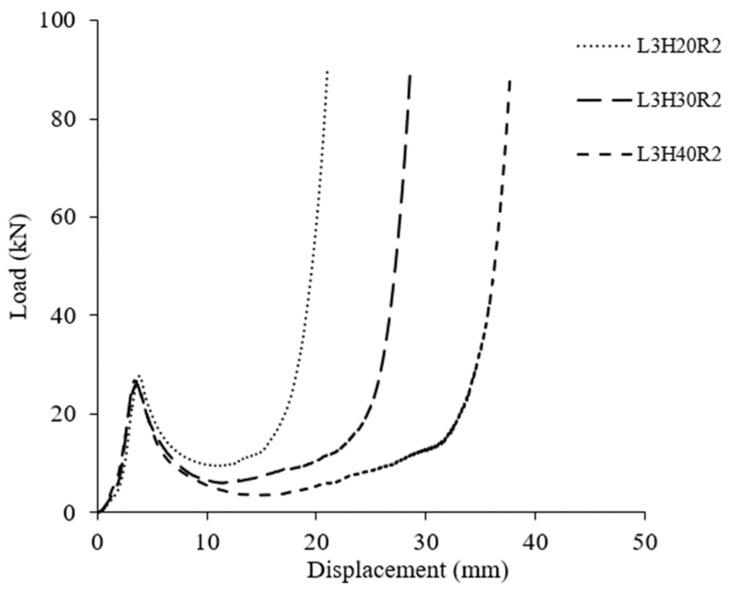
Typical load-displacement curve for all core height at loading rate of 2 mm/min.

**Figure 7:**
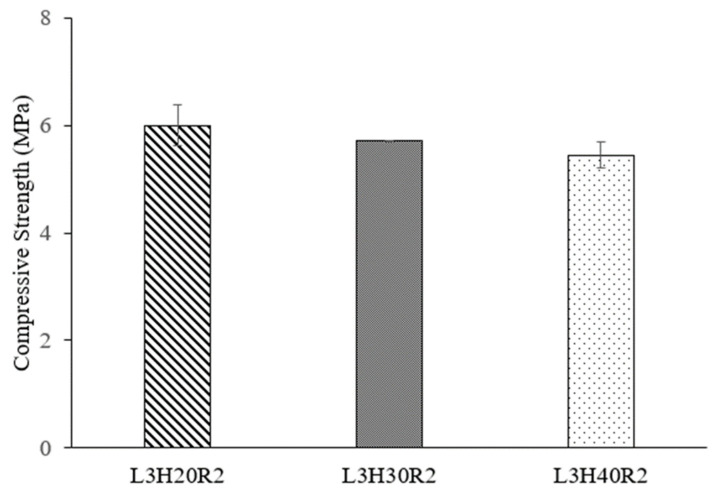
The compressive strength of sample with different height at a rate of 2 min/mm.

Here, the attention is to investigate the effect of core height on compression strength. Figure 6 shows the variation of load-displacement of different core heights at a crosshead displacement of 2 mm/min. In Figure 6, all samples show a similar trend, with the load increasing until its peak load and a sudden drop of load, then followed by a constant plateau until the structures are fully densified. It can be observed that all three samples offer similar peak values. A comparison of honeycomb structural compressive strengths at different core heights are presented in Figure 7. It is clearly shown that the average compressive strength of each sample shows no significant difference. For example, sample L3H20R2 reached its average compressive strength at 6 MPa, while L3H30R2 and L3H40R2 had compressive strengths of 5.71 MPa and 5.45 MPa, respectively. This can be seen in Figure 6, where all samples reached their peak load at a similar point. On close observation, the values of compressive strength between these three parameters are approximately 5–11%, which is relatively small. However, the onset densification of each sample present significantly different due to the differences in height as well some evidence of decreasing of plateau load as the core height increases.

### 4.3. Effect of Loading Rate on the Compression Strength

**Figure 8:**
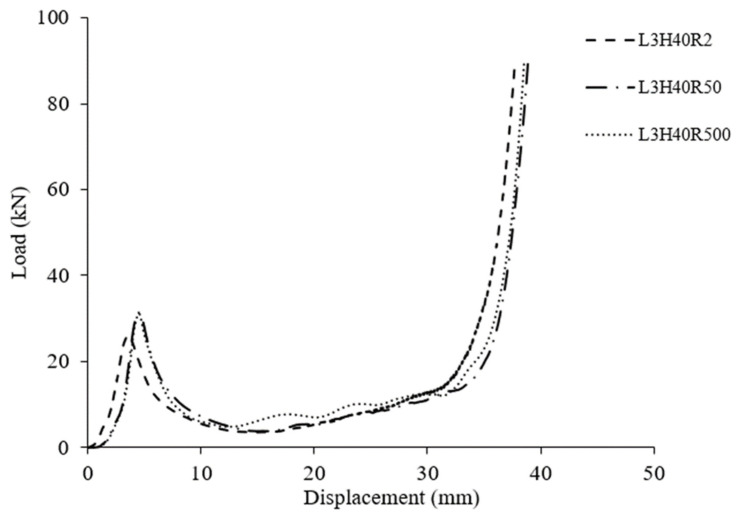
Typical load-displacement curve for all core height of 40 mm at different loading rate.

**Figure 9:**
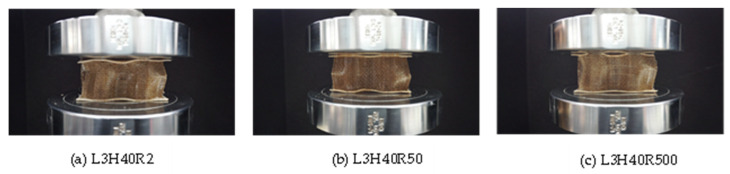
Failure mode of core height of 40 mm with three layers for loading rates of (**a**) 2 mm/min, (**b**) 50 mm/min and (**c**) 500 mm/min.

Furthermore, based on Figure 8, at a core height of 40 mm, all loading rates showed no significant effect in terms of their maximum value, plateau loading and onset densification. The maximum peak value for all samples was approximately 30 kN, and the peak load occurred at a displacement of 5 mm. This is might due to the small difference in loading rate, which had no effect on strength of the structure. However, close monitoring showed that the loading rate of 2 mm/min gave the lowest value compared to the others. Figure 9 present the failure mode of core at 40 mm height under different loading rates taken at 5 mm, and the failure showed a similar mode which was likely due to composite fibre breakage after buckling.

### 4.4. Energy Absorption Capability

The specific energy absorption (SEA) was calculated from the value under the load-displacement graph up to their densification point and divided with the mass of the core. The focus of this part was to investigate the influence of the core height and loading rate. Overall, it can be observed that L3H20R500 outperformed all other samples in this study. The SEA for L3H20R500 offered the highest value at around 12.36 kJ/kg, which was approximately 16% and 9% higher than L3H20R2 and L3H20R50, respectively. Meanwhile, at the same loading rate, the higher core height reduced the SEA value. This was applied to all samples, as can be seen for L3H30 and L3H40. However, at the highest loading rate (R500), the core heights of 30 mm and 40 mm showed similar values. This was due to the very small difference in core height that does not affect the SEA value significantly. By referring to Figure 6, it can be observed that the constant plateau load of L3H30R2 was much higher than L3H40R2. However, the densification points of L3H40R2 achieved a higher displacement, thus making both SEA values around the same in total. One can see that the SEA value exhibited a positive trend, where it increased when the loading rate increased at the same core height. These findings are similar to that stated by Obadimu and Kourousis [36], where the loading rate had a major influence on the energy absorption and deformation of a structure.

**Figure 10:**
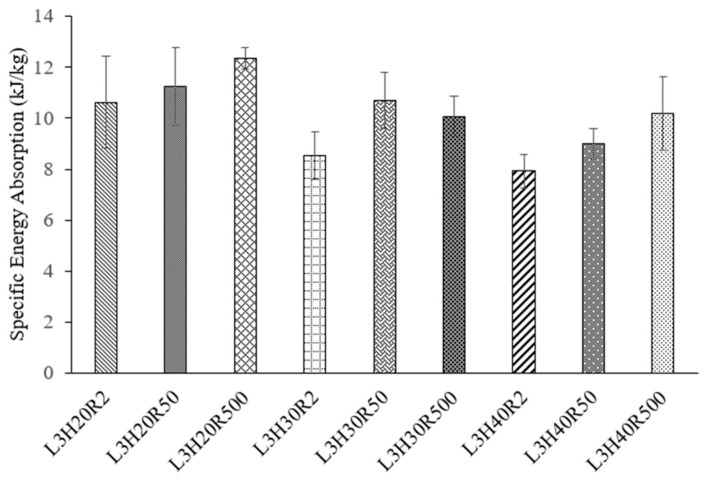
SEA values for kenaf/PLA sandwich structure with different core heights and loading rates.

In terms of a core height effect, L3H20R2 outperformed its counterparts L3H30R2 and L3H40R2 by 24% and 34%, respectively. The observation continues at different rates of 50 mm/min and 500 mm/min. There was a similar trend of decreasing SEA as the core height increased. For example, L3H20R50 had a higher value of SEA compared to L3H30R50 and L3H40R50 by 11% and 25%, respectively. These findings are consistent with those published in [27], which conclude that core height has significant effect on the specific energy absorption (SEA). The overall specific energy absorption values for all samples at different heights and loading rates are summarised in Figure 10.

To understand more on the performance and capabilities of kenaf/PLA honeycomb core sandwich structure, the current data on specific energy absorption was compared to previous work that involved variants of sandwich core structures, as shown in Table 2. The highlighted sample is L3H20R500, since it had the highest SEA value obtained, which was 12.36 kJ/kg. L3H20R500 was 53% higher compared to 20 mm square core Flax/PLA filled with F150 foam, as recorded by Alsubari et al. [22]. Moreover, L3H20R500 had a 7-fold higher value of SEA than the data recorded by Al-Azad and Kamal [37] and 2-fold greater than Han et al. [38]. It should be noted that Alsubari [23] and Al-Azad and Kamal [37] used the same category of natural fibre-based material while Han [38] used aluminum-based material. However, it should be noted that the current study still underperformed with respect to the Nomex material sandwich structure tested by Zhou et al. [14], where the SEA recorded was 15.24 kJ/kg. This value is about 23% higher compared to sample L3H20R2.

## 5. Conclusions

In this paper, the compression properties of kenaf/PLA sandwich structures have been studied. The effects of their core heights and loading rates were investigated under quasi-static compression tests. The following conclusions can be drawn:

- It was observed that at different core heights and loading rates, the samples showed no significant difference in values of compressive strength. Thus, core height and loading rate do not have any influence in this study.
- It was found that the specific energy absorption (SEA) showed an increasing value as the loading rate increased. The SEA for L3H20R500 offered the highest value of around 12.36 kJ/kg, which was approximately 16% and 9% higher than for L3H20R2 (loading rate 2 mm/min) and L3H20R50 (loading rate 50mm/min).
- The increase of core height reduced the SEA. L3H20R50 (core height of 20 mm) had a higher value of SEA compared to L3H30R50 (core height of 30 mm) and L3H40R50 (core height of 40 mm) by 11% and 25%, respectively.
- The sandwich structure made of kenaf/PLA composites was mostly found to fail due to buckling and fibre breakage.

## Figures and Tables

**Table 1:** Dimensions of the honeycombs core structures.

Sample ID	Core Height, H (mm)	Nominal Thickness, T_n_ (mm)	$\mathrm{Density},\;\overset{-}{\rho }$ (kg/m^3^)	Loading Rate, R (mm/min)	Dimension, Width (w) × Length (L) (mm)
L3H20R2	20	2	192.51	2	14 × 85
L3H20R50				50	
L3H20R500				500	
L3H30R3	30		185.38	2	
L3H30R50				50	
L3H30R500				500	
L3H40R2	40		181.82	2	
L3H40R50				50	
L3H40R500				500	

**Table 2:** Comparison of compression behavior of kenaf/PLA composite with published studies.

Type of Core	Type of Material	Relative Density	Compressive Strength (MPa)	SEA (kJ/kg)	Ref
Honeycomb	Kenaf/PLA (Core height: 20 mm, Loading rate: 500 mm/min)	-	6.23	12.36	Current study
	Kenaf/PLA (Core height: 23 mm, Loading rate: 500 mm/min)	-	5.06	10.08	
	Kenaf/PLA (Core height: 40 mm, Loading rate 500 mm/min)	-	6.33	10.2	
Square core	Flax/PLA (Cell size: 20 mm filled with F150 foam)	0.55	8	8.5	[22]
	Flax/PLA (Cell size: 30 mm filled with F150 foam)	0.58	6.55	8.05	
	Flax/PLA (Cell size: 40 mm filled with F150 foam)	0.62	4.9	7.90	
XX type lattice core	MDF + birch (Core diameter: 8 mm)	-	1.76	323.52	[26]
	Plywood + birch (Core diameter: 8 mm)	-	1.94	422.06	
Honeycomb core	Corn starch (Cell wall thickness: 0.145 mm, Core thickness: 90 mm)	-	-	0.99	[37]
	Corn starch (Cell wall thickness: 0.29 mm, Core thickness: 90 mm)	-	-	1.62	
Honeycomb	Nomex	-	-	15.24	[14]
Corrugated and Trapezoidal honeycomb core	Hybrid Aluminium of Al-3003-H18 and Al-3003-H24 (Corrugated core plate thickness: 0.2 mm)	-	5.74	6.85	[38]
	Hybrid Aluminium of Al-3003-H18 and Al-3003-H24 (Corrugated core plate thickness: 0.4 mm)	-	8.14	6.76	
	Hybrid Aluminium of Al-3003-H18 and Al-3003-H24 (Corrugated core plate thickness: 0.7 mm)	-	11.07	6.5	

## Data Availability

Not applicable.
